# Trends in Sunscreen Use Among US Middle and High School Students, 2007-2019

**DOI:** 10.7759/cureus.16468

**Published:** 2021-07-18

**Authors:** Geethanjali Rajagopal, Rachna Talluri, Valerie S Chuy, An-Lin Cheng, Lawrence Dall

**Affiliations:** 1 Internal Medicine, University of Missouri Kansas City School of Medicine, Kansas City, USA; 2 Biomedical and Health Informatics, University of Missouri Kansas City School of Medicine, Kansas City, USA

**Keywords:** adolescents, pediatrics, sunscreen, skin cancer, students

## Abstract

Objective: The purpose of this project is to analyze trends in sunscreen usage among middle and high school students from the Centers of Disease Control’s National Youth Risk Behavior Surveys (YRBS) from the years 2007 to 2019.

Methods: Data from the 2007-2019 National YRBS were analyzed. YRBS is a cross-sectional survey of health risk behaviors among middle and high school students (grades 6-12) in the United States. Students were asked questions regarding sunscreen usage and demographic information, including age, race/ethnicity, and gender.

Results: From 2007 to 2019, the average mean sunscreen usage for all students increased by 4% between every consecutive year studied. Mean sunscreen usage among all racial groups studied, other than Native Hawaiian or Other Pacific Islanders, increased. Females were 92.7% more likely to use sunscreen than males each year. The mean sunscreen usage decreased by 5% with an increase in a student’s age.

Conclusions: Though overall sunscreen use in adolescents increased over the time period from 2007 to 2019, sunscreen use still remains limited due to a variety of factors possibly including cost, tanning, and different socio-cultural perceptions of sunscreen.

## Introduction

Per the Centers for Disease Control (CDC), rates of incidence of cutaneous melanomas have been rising from 1999 to 2017 [1]. A 2018 article published in JAMA Dermatology found that self-reported usage of sunscreen during childhood and adolescence is associated with a reduced risk of cutaneous melanoma among young adults [2]. Regular usage of sunscreen also protects against squamous cell carcinoma of the skin and photoaging. While not statistically significant, studies suggest reduced rates of basal cell carcinoma of the skin with regular sunscreen use [3]. Previous studies regarding adolescent attitudes and knowledge of sun safety demonstrate an alarming trend. A 2014 study of Florida adolescents demonstrated that 63% of participants were not aware of peak hours of UV exposure and 62% “sometimes,” “rarely,” or “never” apply sunscreen. Eighty percent of participants believed that a suntan looked healthy, while only 67% of participants felt that they were at risk for skin cancer development [4]. With rising rates of squamous cell carcinoma and a clear ambivalence towards sunscreen use amongst teenagers, we sought to examine the frequency of sunscreen use amongst US adolescents. The purpose of this project was to analyze trends in sunscreen usage among middle and high school students from CDC’s National Youth Risk Behavior Surveys (YRBS) from the years 2007 to 2019.

## Materials and methods

The YRBS is a cross-sectional survey of health risk behaviors administered to middle and high school students (grades 6-12) attending private and public schools across the United States. Students were categorized as 12 years and younger, 13, 14, 15, 16, 17, and 18 years and older. Students voluntarily completed the survey during school, and the results were kept anonymous. The questionnaire included 80-100 multiple choice questions assessing different health risk behaviors (drug/tobacco use, diet, exercise, sunscreen use, etc.). Questionnaires were administered to schools biennially. Parental permission was obtained as per school regulations. All questions on the YRBS questionnaire were deemed statistically reliable [5]. CDC has received Institutional Review Board clearance for YRBS reports and data. Our study was an analysis of publicly available data provided by the CDC and did not include identification of individuals who participated in the survey. Therefore, this study did not require IRB review. 

This was a retrospective database analysis of YRBS records from the years 2007, 2009, 2011, 2013, and 2019. In these years, students were asked “When you are outside for more than one hour on a sunny day, how often do you wear sunscreen with an Sun Protection Factor (SPF) of 15 or higher?” Responses included “never,” “rarely,” “sometimes,” “most of the time,” and “always.” In the 2015 and 2017 YRBS questionnaires, students were not asked about sunscreen use. The five responses to the sunscreen questions were assigned a number ranging from 1 to 5 (never: 1, rarely: 2, sometimes: 3, most of the time: 4, always: 5). Students were also asked demographic questions, including race/ethnicity, age, and gender. For every age, race/ethnicity, and gender, mean sunscreen usage (from 1 to 5) was calculated for every year. Graphs were created to demonstrate trends for average sunscreen usage over time. A proportional odds model (Table 1) was conducted to estimate and test the linear effect of each year between 2007 and 2019 on sunscreen usage while accounting for age, gender, and race/ethnicity (see reading materials by Agresti [6] and Hosmer and Lemeshow [7] for an explanation of the proportional odds model).

**Table 1:** Proportional Odds Model Testing Linear Effect of Year on Sunscreen Age

		95% Confidence Interval		
Factor	Odds Ratio	Lower Limit	Upper Limit	P-Value
Year	1.044	1.04	1.049	<0.0001
Age	0.947*	0.935	0.959	<0.0001
Gender (Compared against Females)	1.927	1.867	1.989	<0.0001
American Indian/Alaskan Native vs Hispanic/Latino	0.996	0.871	1.138	<0.0001
Asian vs Hispanic/Latino	1.81	1.666	1.965	<0.0001
Black vs Hispanic/Latino	0.403	0.382	0.424	<0.0001
Native Hawaiian or Other Pacific Islander vs Hispanic/Latino	1.117	0.936	1.334	<0.0001
White vs Hispanic/Latino	1.87	1.802	1.94	<0.0001

## Results

**Figure 1:**
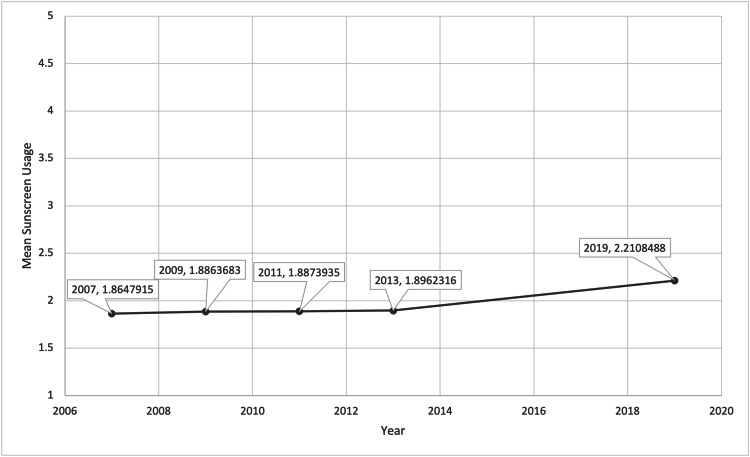
Mean Sunscreen Usage Over the Years 2007 to 2019

**Figure 2:**
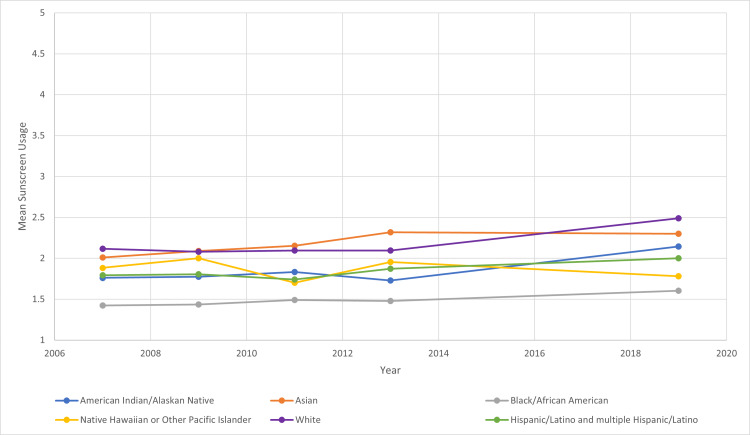
Mean Sunscreen Usage Trends by Race/Ethnicity Over the Years 2007 to 2019

**Figure 3:**
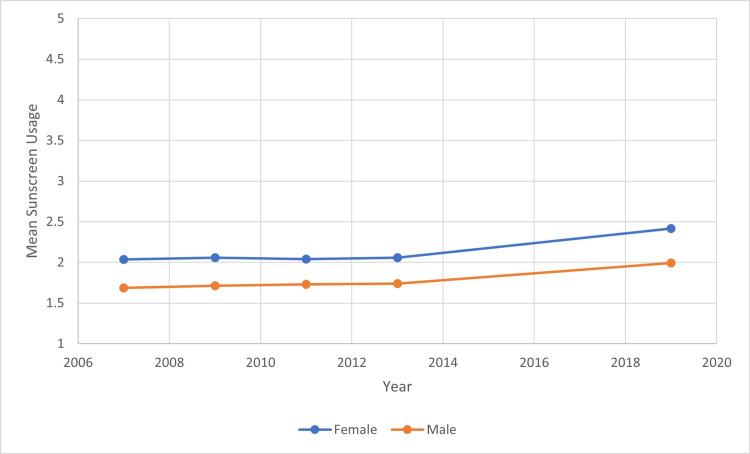
Mean Sunscreen Usage Trends by Gender Over the Years 2007 to 2019

**Figure 4:**
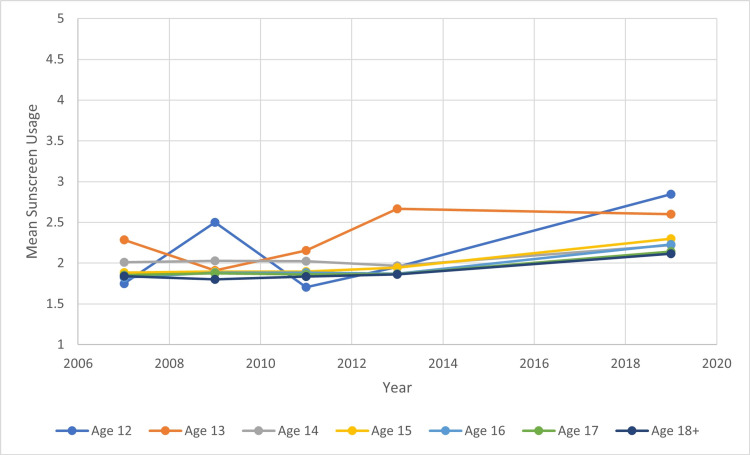
Mean Sunscreen Usage Trends by Age Over the Years 2007 to 2019

The total number of participating students in the surveys each year ranged from 13,513 to 16,410. Using the proportional odds model, we were able to determine the relationship between sunscreen usage and year, age, gender, and race/ethnicity. From 2007 to 2019, the average mean sunscreen usage for all students increased by 4% between every consecutive year studied (Figure 1). Over the course of 2007 to 2019, the mean sunscreen usage of White, Asian, American Indian/Alaskan Native, Hispanic/Latino, and Black/African Americans increased overall. There was an overall decrease in mean sunscreen usage among Native Hawaiian or Other Pacific Islanders (Figure 2). Average mean sunscreen use from 2007 to 2019 was highest for White students (mean 2.175) followed by Asian students (2.173), Native Hawaiian or Other Pacific Islander students (mean 1.864), American Indian/Alaskan Native (mean 1.848), Hispanic/Latino students (mean 1.842), and Black/African American students (mean 1.486). When comparing sunscreen usage between male and female participants, there was a statistically significant higher mean sunscreen usage in females in comparison to males in all years surveyed (Figure 3). Females were 92.7% more likely to use sunscreen than males. The mean sunscreen usage decreased by 5% with each one year increase in a student’s age (Figure 4).

## Discussion

Two previous articles utilizing YRBS data have analyzed trends in sunscreen usage among middle and high school students from 1999 to 2003 and 1999 to 2009, respectively [8,9]. The latter study found that there was an overall decrease in reported sunscreen use among students between the years 1999 and 2009 [9]. We found that there was an increase in overall reported sunscreen use between the years 2007 and 2019. One reason for this increase could be the increased use of social media promoting awareness of sunscreen. In a study done in Australia, researchers looked at tweets posted over the summer of 2018 and 2019 that had the keyword "sunscreen." Results showed that Australians used Twitter as a means to share advice related to sun-related cancers and sunscreen usage [10]. Another study done by William Paterson University and Columbia University studied the exposure of adolescents and young adults to skin cancer information and preventative measures [11]. The study found that Instagram content focused most on the prevention of skin cancer. Instagram posts that covered skin cancer prevention discussed the role of sun exposure in the development of skin cancer and the use of sunscreen and protective gear. 

We found that sunscreen use decreased with an increase in age. One explanation could be an increased interest in tanning outdoors among older adolescents. One study showed that tanning practices increased significantly between 14 and 17-year-olds in a graded fashion. The study found that increased tanning practices and positive attitudes toward tanning were associated with more sporadic sunscreen use compared to regular sunscreen use [12].

There is a significant difference between sunscreen use in females and males. One explanation for the increased use of sunscreen in females could be the incorporation of sunscreen in make-up and skincare products, much of which are currently marketed toward females. A 2003 study that analyzed gender differences in sunscreen use found that females were more likely to use sunscreen to enhance their cosmetic appearance (prevent wrinkles, sunburns, etc.). This study also reported that sunscreen use in males was typically limited to times with extended sun exposure versus in females; sunscreen use was utilized in a broader range of settings [13].

The previous articles studying sunscreen usage using YRBS data primarily investigated trends among people of White, Black/African American, and Hispanic/Latino backgrounds [8,9]. With increasing racial diversity in America, it is valuable to examine sunscreen usage trends in other racial backgrounds as well. We found a significant relationship between race/ethnicity and sunscreen usage. Previous studies have found that differences in sunscreen usage between races/ethnicities could be attributed to varying perceptions and education regarding skin cancer risk. One study of high school students of different ethnic and racial backgrounds found that White participants had the greatest knowledge on sun protection measures in comparison to other racial groups, followed by Asian participants. Black participants had the least knowledge regarding sun protection measures [14]. A study looking at skin cancer knowledge among Black and Hispanic adults found that a majority of participants believed that they were at lower skin cancer risk due to being of darker skin tone or having no family history of skin cancer. Participants also endorsed childhood discussion regarding sun protection to be primarily focused on the protection of appearance rather than protection against skin cancer [15]. Another study showed that Native Americans have been shown to have poor sunscreen usage as they believed that they were not likely to develop skin cancer [16].

Though there was an overall increase in reported sunscreen use from 2007 to 2019, the average reported sunscreen use still fell between the range of 1 to 3 (participants reporting “sometimes” to “never” using sunscreen). There are still many barriers to proper sunscreen use. An institutional survey of patients at a dermatology clinic found that 33.7% of participants identified dislike of the feel or appearance of sunscreen as a barrier to using. Other barriers identified were time constraints (15.3%) and cost (16.4%) [17].

The importance of sunscreen usage for skin cancer prevention has been heavily documented in the medical field. The primarily low utilization of sunscreen across all populations in this study demonstrates the need for increased education among the general adolescent population. The utilization of sunscreen use could be improved by the use of social media campaigns, as adolescents have come to spend an increasing amount of time on these platforms. Integration of sunscreen information and skin cancer awareness in school health classes could also create a change in sunscreen habits among a larger group of adolescents. This outreach must also emphasize the risk of skin cancer among all racial groups, as previous studies have shown that absent or reduced risk of skin cancer is a common misperception in people of color [15,16].

Some limitations of this study should be considered. The YRBS questionnaires are administrated during February, March, and April, which could ignore possible seasonal patterns in sunscreen usage. Additionally, investigators are unable to conclude if a student utilized sunscreen more or less during certain times of the year, or began using sunscreen recently, but consistently, or if a student had been using sunscreen for many years. Another limitation of this study was that other types of protection from the sun were not recorded, including visors, long sleeves, avoiding the sun, staying in the shade, etc. Concurrently, the study did not specify the duration that each student spent in the sun or the amount of time between students reapplying sunscreen. Quantification of terms like “rarely using sunscreen” or “sometimes using sunscreen” could have aided in strengthening this study. Further investigations of this study could include seeing if the trends of this study correlate to YRBS’s data in 2021, especially as more students are splitting their time between in-person and online school due to the COVID-19 pandemic. Students may have spent less time in direct sunlight in 2020-2021, and, as a result, decreased their sunscreen usage. Individual students within each age group could also be interviewed regarding their reasonings for using sunscreen “never,” “rarely,” “sometimes,” “most of the time,” or “always.” As a result, investigators will gain more insight into whether the decision to wear sunscreen was influenced by cultural, educational, or other social factors. Further questioning regarding other types of sunscreen protection, the specific SPF numbers, and seasonal changes in sunscreen usage can also be obtained.

## Conclusions

Regular sunscreen use is known to decrease rates of skin cancer, including melanoma and squamous cell carcinoma. We analyzed CDC’s YRBS questionnaires to investigate trends in sunscreen usage among middle and high school students in the United States from 2007 to 2019. Significant differences in sunscreen were found between students of different ages, races, gender, and year of survey administration. Sunscreen use increased overall from previous studies, possibly due to an increase in social media awareness. Sunscreen use still remains limited due to a variety of factors possibly including cost, tanning, and different socio-cultural perceptions of sunscreen.
